# 1,2-Bis[5-(2,2′-dicyano­vinyl)-2-*n*-pentyl-3-thien­yl]-3,3,4,4,5,5-hexa­fluoro­cyclo­pent-1-ene: a new photochromic diaryl­ethene compound

**DOI:** 10.1107/S160053680800202X

**Published:** 2008-01-25

**Authors:** Min Li, Shou-Zhi Pu, Cong-Bin Fan, Zhang-Gao Le

**Affiliations:** a Key Laboratory of Nuclear Resources and Environment of the Ministry of Education, East China Institute of Technology, Fuzhou 344000, People’s Republic of China; b Jiangxi Key Laboratory of Organic Chemistry, Jiangxi Science & Technology Normal University, Nanchang 330013, People’s Republic of China

## Abstract

The title compound, C_31_H_26_F_6_N_4_S_2_, is a new photochromic dithienylethene with dicyano­vinyl subsitituents. In the crystal structure, the mol­ecule adopts a photoactive anti­parallel conformation, with two *n*-pentyl groups located on opposite sides of the cyclo­pentene ring. The cyclo­pentene ring assumes an envelope conformation. The distance between the two reactive C atoms on the thio­phene rings is 3.834 (7) Å. One of the *n*-pentyl groups is disordered over two positions; the site occupancy factors are *ca* 0.7 and 0.3.

## Related literature

For general background, see: Gilat *et al.* (1993 ▶, 1995 ▶); Irie (2000 ▶); Pu *et al.* (2003 ▶, 2005 ▶); Tian & Yang (2004 ▶); Yamaguchi & Irie (2006 ▶); Zheng *et al.* (2007 ▶). For related structures, see: Kobatake *et al.* (2004 ▶); Woodward & Hoffmann (1970 ▶).
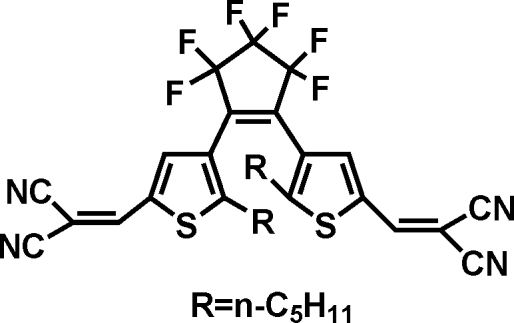


## Experimental

### 

#### Crystal data

C_31_H_26_F_6_N_4_S_2_
*M* *_r_* = 632.68
Triclinic, 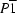

*a* = 9.2500 (12) Å
*b* = 12.3670 (16) Å
*c* = 15.596 (2) Å
α = 67.730 (2)°
β = 85.482 (2)°
γ = 72.804 (2)°
*V* = 1576.1 (4) Å^3^
*Z* = 2
Mo *K*α radiation
μ = 0.23 mm^−1^
*T* = 291 (2) K
0.29 × 0.21 × 0.16 mm

#### Data collection

Bruker SMART APEXII CCD area-detector diffractometer
Absorption correction: multi-scan (*SADABS*; Sheldrick, 1996 ▶) *T*_min_ = 0.932, *T*_max_ = 0.963
12153 measured reflections
5828 independent reflections
3782 reflections with *I* > 2s˘*I*)
*R*_int_ = 0.026

#### Refinement

*R*[*F* ^2^ > 2σ(*F* ^2^)] = 0.049
*wR*(*F* ^2^) = 0.142
*S* = 1.03
5828 reflections
384 parameters
14 restraints
H-atom parameters constrained
Δρ_max_ = 0.41 e Å^−3^
Δρ_min_ = −0.26 e Å^−3^

### 

Data collection: *APEX2* (Bruker, 2004 ▶); cell refinement: *SAINT* (Bruker, 1997 ▶); data reduction: *SAINT*; program(s) used to solve structure: *SHELXTL* (Sheldrick, 2008 ▶); program(s) used to refine structure: *SHELXTL*; molecular graphics: *SHELXTL*; software used to prepare material for publication: *SHELXTL*.

## Supplementary Material

Crystal structure: contains datablocks I, global. DOI: 10.1107/S160053680800202X/xu2397sup1.cif

Structure factors: contains datablocks I. DOI: 10.1107/S160053680800202X/xu2397Isup2.hkl

Additional supplementary materials:  crystallographic information; 3D view; checkCIF report

## Figures and Tables

**Fig. 1.**
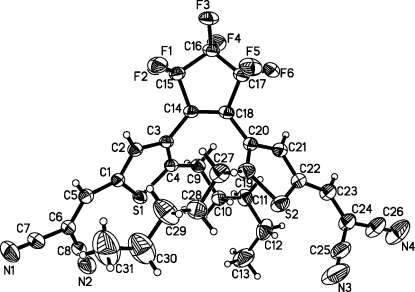
The molecular structure of (Ia) with 35% probability ellipsoids, showing the atomic numbering scheme. The minor disordered component has been omitted for clarity.

**Fig. 2.**
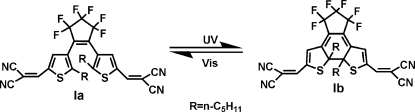
The tautomerism of the title compound and the closed-ring tautomer (Ib).

**Fig. 3.**
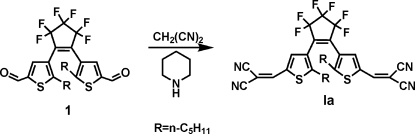
The formation of the title compound.

## supplementary crystallographic information

### Comment

Photochromism has attracted considerable attention because of its potential application to photonic devices, such as optical memories and optical switches (Yamaguchi & Irie, 2006). Among various types of photochromic compounds, diarylethenes bearing two thiophene-derived groups have received the most attention because of their excellent fatigue resistant and outstanding thermally irreversible photochromic performance (Irie, 2000; Tian & Yang, 2004). From the viewpoint of applications to optical memory media and full color diaplays, it is desired to develop photochromic compounds that have sensitivity in the wavelength region of 650–830 nm (Irie, 2000; Gilat *et al.*, 1993, 1995). We have previously reported three dithienylethenes with dicyano subsitituent: 1,2-bis[2-methyl-5-(2,2'-dicyanovinyl)-3-thienyl]-3,3,4,4,5,5- hexafluorocyclopent-1-ene, 2-ethyl analog and 2-butyl analog (Pu *et al.*, 2003, 2005; Zheng *et al.*, 2007), which have relatively long wavelength absorption spectrum (> 700 nm). In order to investigate systematically the substituent effect at the 2-position of the thiophene rings of diarylethenes on their photochemical properties, we have now synthesized the title photochromic diarylethene, (Ia) (Scheme 1), and its structure is presented here.

The molecular structure of the title compound (Ia) (Fig. 1) shows a photoactive anti-parallel conformation. The two n-pentyl groups are *trans* directed with respect to the central cyclopentene ring. Such a configuration is crucial for the compound to exhibit photochromic and photoinduced properties (Woodward & Hoffmann, 1970). The central cyclopentene ring assumes an envelope conformation. The distance between the two reactive C atoms (C4 and C19) is 3.834 (7) Å. This distance indicates the crystal can undergo photochromism to generate the closed isomer of diarylethene (Ib), because photochromic reactivity usually appears when the distance between the reactive C atoms is less than 4.2 Å (Kobatake *et al.*, 2004). Crystal of (Ia) showed photochromic reaction coincident with the theoretical analysis. The colorless crystal of (Ia) turned to green upon irradiation with 365 nm UV light. When the green crystal were dissolved in hexane, the solution turned to green, and the absorption maximum was observed at 750 nm, which is the identical with that found in its closed-ring tautomer (Ib) (Scheme 2). The green color disappeared upon irradiation with appropriate wavelength visible light and the absorption spectrum of the solution containing the colorless crystal were the same as that found for the open-ring tautomer (Ia) with the absorption maximum at 356 nm.

### Experimental

The title compound, (Ia), was synthesized by the Knoevenagel condensation reaction of diarylethene 1,2-bis(2-n-heptyl-5-formyl-3-thienyl)perfluorocyclopentene (1) (Scheme 3). First, compound (1) was prepared according to the procedure described in the previous paper (Zheng *et al.*, 2007). Then to a stirred solution of compound (1) (0.1 g, 0.19 mmol) and malonodinitrile (0.025 g, 0.35 mmol) in anhydrous ethanol (5 ml), a very small quantity of piperidine was added dropwise at room temperature. The reaction mixture was stirred overnight at 323 K. Finally, the title compound was produced in 75.0% yield.

### Refinement

One of n-pentyl groups is disordered over two distinct conformations. The site occupancies were refined and converged to 0.656:0.344. The H atoms were positioned theoretically and allowed to ride on their parent atoms in the final refinement, with C—H = 0.93–0.97 Å and *U*_iso_(H) =1.2*U*_eq_(C) or 1.5*U*_eq_(methyl C). The methyl groups were treated as rigid groups and allowed to rotate about the C—C bond. Atomic displacement parameters for the disordered components were restrained.

### Crystal data

**Table:**

C_31_H_26_F_6_N_4_S_2_	*Z* = 2
*M**_r_* = 632.68	*F*_000_ = 652
Triclinic, *P*1	*D*_x_ = 1.333 Mg m^−^^3^
Hall symbol: -p 1	Melting point: 404.2 K
*a* = 9.2500 (12) Å	Mo *K*α radiation λ = 0.71073 Å
*b* = 12.3670 (16) Å	Cell parameters from 2550 reflections
*c* = 15.596 (2) Å	θ = 2.5–21.8º
α = 67.730 (2)º	µ = 0.23 mm^−^^1^
β = 85.482 (2)º	*T* = 291 (2) K
γ = 72.804 (2)º	Block, colourless
*V* = 1576.1 (4) Å^3^	0.29 × 0.21 × 0.16 mm

### Data collection

**Table:**

Bruker SMART APEXII CCD area-detector diffractometer	5828 independent reflections
Radiation source: fine-focus sealed tube	3782 reflections with *I* > 2s˘*I*)
Monochromator: graphite	*R*_int_ = 0.026
*T* = 291(2) K	θ_max_ = 25.5º
φ and ω scans	θ_min_ = 2.5º
Absorption correction: multi-scan(SADABS; Sheldrick, 1996)	*h* = −11→11
*T*_min_ = 0.932, *T*_max_ = 0.963	*k* = −14→14
12153 measured reflections	*l* = −18→18

### Refinement

**Table:**

Refinement on *F*^2^	Secondary atom site location: difference Fourier map
Least-squares matrix: full	Hydrogen site location: inferred from neighbouring sites
*R*[*F*^2^ > 2σ(*F*^2^)] = 0.049	H-atom parameters constrained
*wR*(*F*^2^) = 0.142	*w* = 1/[σ^2^(*F*_o_^2^) + (0.0613*P*)^2^ + 0.4466*P*] where *P* = (*F*_o_^2^ + 2*F*_c_^2^)/3
*S* = 1.03	(Δ/σ)_max_ < 0.001
5828 reflections	Δρ_max_ = 0.41 e Å^−^^3^
384 parameters	Δρ_min_ = −0.26 e Å^−^^3^
14 restraints	Extinction correction: none
Primary atom site location: structure-invariant direct methods	

### Special details

**Table:**

Experimental. The structure of (Ia) was confirmed by melting point, NMR, MS, IR, elemental analysis. (m.p.: 404.2 K). ^1^H NMR (400 MHz, CDCl_3_): δ 0.87 (t, 6H, J=7.0 Hz), δ 1.23 (m, 4H), δ 1.43 (m, 4H), δ 2.34 (t, 4H, J=7.8 Hz), δ 7.64 (s, 2H), δ 7.79 (s, 2H). ^13^C NMR (100 MHz,CDCl_3_): δ 113.82, 22.24, 29.91, 30.82, 31.34, 79.87, 112.47, 113.15, 125.31, 133.80, 137.42, 149.55, 159.50. MS m/*z* (*M*^+^) 631.0 (–H). IR (KBr, cm^-1^): 746, 929, 1139, 1276, 1436, 1575, 2225, 2931. Anal. Calcd for C_31_H_26_F_6_N_4_S_2_ (%): Calcd C, 58.85; H, 4.14; N, 8.86. Found C, 58.92; H, 3.72; N, 8.53.
Geometry. All e.s.d.'s (except the e.s.d. in the dihedral angle between two l.s. planes) are estimated using the full covariance matrix. The cell e.s.d.'s are taken into account individually in the estimation of e.s.d.'s in distances, angles and torsion angles; correlations between e.s.d.'s in cell parameters are only used when they are defined by crystal symmetry. An approximate (isotropic) treatment of cell e.s.d.'s is used for estimating e.s.d.'s involving l.s. planes.
Refinement. Refinement of *F*^2^ against ALL reflections. The weighted *R*-factor *wR* and goodness of fit *S* are based on *F*^2^, conventional *R*-factors *R* are based on *F*, with *F* set to zero for negative *F*^2^. The threshold expression of *F*^2^ > σ(*F*^2^) is used only for calculating *R*-factors(gt) *etc*. and is not relevant to the choice of reflections for refinement. *R*-factors based on *F*^2^ are statistically about twice as large as those based on *F*, and *R*- factors based on ALL data will be even larger.

### Fractional atomic coordinates and isotropic or equivalent isotropic displacement parameters (Å^2^)

**Table:**

	*x*	*y*	*z*	*U*_iso_*/*U*_eq_	Occ. (<1)
C27	0.0741 (4)	0.5366 (3)	0.1942 (2)	0.0749 (11)	0.656 (4)
H27A	−0.0184	0.5762	0.1559	0.090*	0.656 (4)
H27B	0.0469	0.5193	0.2584	0.090*	0.656 (4)
C28	0.1730 (4)	0.6205 (3)	0.1681 (3)	0.0867 (15)	0.656 (4)
H28A	0.1966	0.6409	0.1032	0.104*	0.656 (4)
H28B	0.2673	0.5799	0.2047	0.104*	0.656 (4)
C29	0.0917 (4)	0.7363 (3)	0.1854 (3)	0.131 (3)	0.656 (4)
H29A	0.0013	0.7809	0.1454	0.158*	0.656 (4)
H29B	0.0622	0.7163	0.2494	0.158*	0.656 (4)
C30	0.2013 (6)	0.8128 (4)	0.1645 (4)	0.167 (4)	0.656 (4)
H30A	0.2968	0.7652	0.1987	0.201*	0.656 (4)
H30B	0.2203	0.8424	0.0988	0.201*	0.656 (4)
C31	0.1251 (9)	0.9181 (5)	0.1947 (5)	0.194 (4)	0.656 (4)
H31A	0.0851	0.9904	0.1411	0.291*	0.656 (4)
H31B	0.1976	0.9313	0.2275	0.291*	0.656 (4)
H31C	0.0441	0.8994	0.2346	0.291*	0.656 (4)
C27'	0.1072 (4)	0.5322 (3)	0.2057 (2)	0.0749 (11)	0.344 (4)
H27C	0.0193	0.5294	0.2439	0.090*	0.344 (4)
H27D	0.1882	0.5305	0.2425	0.090*	0.344 (4)
C28'	0.0690 (7)	0.6517 (3)	0.1229 (2)	0.0867 (15)	0.344 (4)
H28C	0.0012	0.6481	0.0803	0.104*	0.344 (4)
H28D	0.1611	0.6620	0.0909	0.104*	0.344 (4)
C29'	−0.0053 (5)	0.7624 (3)	0.1489 (5)	0.131 (3)	0.344 (4)
H29C	−0.0661	0.8272	0.0962	0.158*	0.344 (4)
H29D	−0.0725	0.7406	0.1994	0.158*	0.344 (4)
C30'	0.1095 (5)	0.8093 (4)	0.1779 (3)	0.167 (4)	0.344 (4)
H30C	0.2039	0.7449	0.1988	0.201*	0.344 (4)
H30D	0.0716	0.8361	0.2285	0.201*	0.344 (4)
C31'	0.1362 (10)	0.9185 (5)	0.0923 (4)	0.194 (4)	0.344 (4)
H31D	0.2024	0.8876	0.0509	0.291*	0.344 (4)
H31E	0.1814	0.9654	0.1128	0.291*	0.344 (4)
H31F	0.0410	0.9693	0.0604	0.291*	0.344 (4)
S1	0.54961 (8)	0.43367 (6)	0.36166 (5)	0.0514 (2)	
S2	0.20704 (10)	0.42399 (7)	0.07217 (5)	0.0710 (3)	
F1	0.0147 (2)	0.34601 (17)	0.51006 (14)	0.0894 (6)	
F2	0.2160 (2)	0.19831 (18)	0.57122 (12)	0.0842 (6)	
F3	−0.0882 (2)	0.16734 (18)	0.52584 (13)	0.0860 (6)	
F4	0.1357 (2)	0.04837 (16)	0.52485 (12)	0.0781 (5)	
F5	−0.10646 (19)	0.26982 (19)	0.34769 (14)	0.0889 (6)	
F6	0.0564 (2)	0.10029 (17)	0.35752 (12)	0.0806 (6)	
N1	0.4135 (3)	0.9211 (3)	0.3802 (2)	0.0910 (9)	
N2	0.7517 (4)	0.6262 (3)	0.3236 (2)	0.0990 (11)	
N3	0.3212 (8)	0.4646 (4)	−0.1383 (3)	0.196 (3)	
N4	0.4655 (6)	0.0932 (4)	−0.1258 (3)	0.1448 (17)	
C1	0.3935 (3)	0.5249 (2)	0.39481 (17)	0.0466 (6)	
C2	0.2763 (3)	0.4737 (2)	0.41302 (18)	0.0500 (6)	
H2	0.1835	0.5082	0.4336	0.060*	
C3	0.3096 (3)	0.3639 (2)	0.39761 (17)	0.0437 (6)	
C4	0.4564 (3)	0.3291 (2)	0.37081 (17)	0.0465 (6)	
C5	0.3828 (3)	0.6419 (2)	0.39756 (18)	0.0526 (7)	
H5	0.2916	0.6792	0.4180	0.063*	
C6	0.4850 (3)	0.7060 (2)	0.37493 (19)	0.0539 (7)	
C7	0.4460 (4)	0.8257 (3)	0.3782 (2)	0.0650 (8)	
C8	0.6336 (4)	0.6618 (3)	0.3462 (2)	0.0644 (8)	
C9	0.5377 (3)	0.2175 (2)	0.35038 (19)	0.0522 (7)	
H9A	0.4696	0.1681	0.3591	0.063*	
H9B	0.6228	0.1695	0.3943	0.063*	
C10	0.5954 (3)	0.2476 (3)	0.2522 (2)	0.0571 (7)	
H10A	0.5106	0.2977	0.2085	0.069*	
H10B	0.6657	0.2950	0.2442	0.069*	
C11	0.6736 (4)	0.1358 (3)	0.2297 (2)	0.0733 (9)	
H11A	0.6025	0.0894	0.2363	0.088*	
H11B	0.7569	0.0847	0.2743	0.088*	
C12	0.7343 (4)	0.1656 (4)	0.1326 (3)	0.1010 (13)	
H12A	0.6530	0.2225	0.0881	0.121*	
H12B	0.7681	0.0916	0.1195	0.121*	
C13	0.8651 (5)	0.2208 (5)	0.1202 (3)	0.1263 (17)	
H13A	0.8292	0.2993	0.1252	0.190*	
H13B	0.9061	0.2298	0.0602	0.190*	
H13C	0.9425	0.1681	0.1673	0.190*	
C14	0.1988 (3)	0.2956 (2)	0.40680 (18)	0.0445 (6)	
C15	0.1214 (3)	0.2522 (3)	0.4965 (2)	0.0559 (7)	
C16	0.0456 (3)	0.1631 (3)	0.4854 (2)	0.0574 (7)	
C17	0.0347 (3)	0.1976 (3)	0.3810 (2)	0.0544 (7)	
C18	0.1484 (3)	0.2668 (2)	0.34216 (18)	0.0464 (6)	
C19	0.1554 (3)	0.4183 (3)	0.1815 (2)	0.0598 (7)	
C20	0.1843 (3)	0.2997 (2)	0.24345 (18)	0.0469 (6)	
C21	0.2443 (3)	0.2151 (3)	0.20115 (19)	0.0535 (7)	
H21	0.2675	0.1312	0.2333	0.064*	
C22	0.2660 (3)	0.2670 (3)	0.10778 (19)	0.0556 (7)	
C23	0.3278 (4)	0.2002 (3)	0.0501 (2)	0.0680 (8)	
H23	0.3486	0.1159	0.0792	0.082*	
C24	0.3606 (4)	0.2403 (3)	−0.0400 (2)	0.0749 (9)	
C25	0.3391 (6)	0.3645 (5)	−0.0945 (3)	0.1073 (15)	
C26	0.4192 (5)	0.1571 (4)	−0.0867 (3)	0.1014 (13)	

### Atomic displacement parameters (Å^2^)

**Table:**

	*U*^11^	*U*^22^	*U*^33^	*U*^12^	*U*^13^	*U*^23^
C27	0.087 (3)	0.0505 (19)	0.069 (2)	−0.0050 (18)	0.001 (2)	−0.0136 (17)
C28	0.114 (5)	0.063 (3)	0.075 (4)	−0.030 (3)	0.000 (3)	−0.013 (3)
C29	0.144 (7)	0.054 (3)	0.185 (7)	−0.022 (4)	−0.012 (5)	−0.034 (4)
C30	0.185 (10)	0.104 (4)	0.209 (7)	−0.039 (5)	−0.068 (7)	−0.043 (4)
C31	0.251 (10)	0.113 (6)	0.232 (10)	−0.054 (6)	−0.027 (9)	−0.071 (6)
C27'	0.087 (3)	0.0505 (19)	0.069 (2)	−0.0050 (18)	0.001 (2)	−0.0136 (17)
C28'	0.114 (5)	0.063 (3)	0.075 (4)	−0.030 (3)	0.000 (3)	−0.013 (3)
C29'	0.144 (7)	0.054 (3)	0.185 (7)	−0.022 (4)	−0.012 (5)	−0.034 (4)
C30'	0.185 (10)	0.104 (4)	0.209 (7)	−0.039 (5)	−0.068 (7)	−0.043 (4)
C31'	0.251 (10)	0.113 (6)	0.232 (10)	−0.054 (6)	−0.027 (9)	−0.071 (6)
S1	0.0473 (4)	0.0541 (4)	0.0602 (4)	−0.0221 (3)	0.0138 (3)	−0.0261 (3)
S2	0.0899 (6)	0.0579 (5)	0.0512 (5)	−0.0162 (4)	0.0068 (4)	−0.0101 (4)
F1	0.0933 (14)	0.0817 (13)	0.1140 (16)	−0.0381 (11)	0.0594 (12)	−0.0601 (12)
F2	0.1018 (14)	0.1093 (15)	0.0517 (11)	−0.0604 (12)	0.0066 (10)	−0.0190 (10)
F3	0.0751 (12)	0.1088 (15)	0.0979 (14)	−0.0552 (11)	0.0483 (11)	−0.0512 (12)
F4	0.0929 (14)	0.0567 (11)	0.0743 (12)	−0.0259 (10)	0.0056 (10)	−0.0104 (9)
F5	0.0490 (10)	0.1086 (16)	0.0957 (14)	−0.0291 (11)	−0.0027 (10)	−0.0177 (12)
F6	0.1067 (15)	0.0806 (13)	0.0835 (13)	−0.0619 (11)	0.0303 (11)	−0.0410 (11)
N1	0.095 (2)	0.0611 (19)	0.126 (3)	−0.0279 (17)	0.0086 (19)	−0.0414 (18)
N2	0.087 (2)	0.115 (3)	0.134 (3)	−0.057 (2)	0.047 (2)	−0.076 (2)
N3	0.404 (9)	0.111 (3)	0.085 (3)	−0.110 (5)	0.091 (4)	−0.040 (3)
N4	0.207 (5)	0.112 (3)	0.085 (3)	0.006 (3)	0.019 (3)	−0.045 (2)
C1	0.0509 (15)	0.0456 (15)	0.0456 (15)	−0.0158 (12)	0.0087 (12)	−0.0194 (12)
C2	0.0470 (15)	0.0519 (16)	0.0545 (16)	−0.0164 (13)	0.0133 (12)	−0.0241 (13)
C3	0.0435 (14)	0.0443 (14)	0.0449 (14)	−0.0172 (12)	0.0094 (11)	−0.0163 (12)
C4	0.0473 (15)	0.0478 (15)	0.0453 (15)	−0.0180 (12)	0.0088 (12)	−0.0166 (12)
C5	0.0564 (17)	0.0524 (16)	0.0495 (16)	−0.0170 (14)	0.0057 (13)	−0.0194 (13)
C6	0.0642 (18)	0.0518 (17)	0.0502 (16)	−0.0238 (15)	0.0040 (14)	−0.0189 (13)
C7	0.069 (2)	0.056 (2)	0.073 (2)	−0.0264 (16)	0.0053 (16)	−0.0214 (16)
C8	0.075 (2)	0.067 (2)	0.069 (2)	−0.0396 (18)	0.0200 (17)	−0.0336 (17)
C9	0.0472 (15)	0.0509 (16)	0.0614 (17)	−0.0162 (13)	0.0104 (13)	−0.0240 (14)
C10	0.0470 (16)	0.0665 (19)	0.0635 (18)	−0.0164 (14)	0.0082 (13)	−0.0315 (15)
C11	0.0623 (19)	0.083 (2)	0.093 (2)	−0.0200 (17)	0.0190 (18)	−0.056 (2)
C12	0.073 (2)	0.154 (4)	0.098 (3)	−0.020 (3)	0.015 (2)	−0.081 (3)
C13	0.120 (4)	0.169 (5)	0.098 (3)	−0.049 (3)	0.045 (3)	−0.061 (3)
C14	0.0406 (14)	0.0417 (14)	0.0501 (15)	−0.0137 (11)	0.0121 (11)	−0.0165 (12)
C15	0.0564 (17)	0.0581 (18)	0.0546 (17)	−0.0215 (15)	0.0159 (14)	−0.0216 (14)
C16	0.0505 (17)	0.0591 (19)	0.0659 (19)	−0.0257 (15)	0.0185 (14)	−0.0224 (15)
C17	0.0479 (16)	0.0557 (17)	0.0636 (18)	−0.0227 (14)	0.0120 (14)	−0.0225 (15)
C18	0.0398 (14)	0.0421 (14)	0.0556 (16)	−0.0130 (12)	0.0082 (12)	−0.0166 (13)
C19	0.0637 (18)	0.0515 (17)	0.0560 (17)	−0.0141 (14)	0.0057 (14)	−0.0136 (14)
C20	0.0442 (14)	0.0505 (16)	0.0474 (15)	−0.0171 (12)	0.0048 (12)	−0.0178 (13)
C21	0.0552 (17)	0.0506 (16)	0.0534 (17)	−0.0189 (13)	0.0037 (13)	−0.0157 (13)
C22	0.0589 (17)	0.0581 (18)	0.0500 (17)	−0.0190 (14)	0.0046 (13)	−0.0193 (14)
C23	0.077 (2)	0.064 (2)	0.059 (2)	−0.0168 (17)	0.0060 (16)	−0.0230 (16)
C24	0.089 (2)	0.073 (2)	0.058 (2)	−0.0186 (19)	0.0077 (18)	−0.0249 (18)
C25	0.177 (5)	0.097 (3)	0.061 (2)	−0.059 (3)	0.040 (3)	−0.037 (2)
C26	0.136 (4)	0.092 (3)	0.060 (2)	−0.011 (3)	0.012 (2)	−0.028 (2)

### Geometric parameters (Å, °)

**Table:**

C27—C28	1.504 (2)	N3—C25	1.133 (5)
C27—C19	1.513 (4)	N4—C26	1.142 (5)
C27—H27A	0.9700	C1—C2	1.370 (4)
C27—H27B	0.9700	C1—C5	1.438 (3)
C28—C29	1.520 (2)	C2—C3	1.410 (3)
C28—H28A	0.9700	C2—H2	0.9300
C28—H28B	0.9700	C3—C4	1.380 (3)
C29—C30	1.520 (2)	C3—C14	1.477 (3)
C29—H29A	0.9700	C4—C9	1.501 (3)
C29—H29B	0.9700	C5—C6	1.350 (4)
C30—C31	1.510 (2)	C5—H5	0.9300
C30—H30A	0.9700	C6—C8	1.426 (4)
C30—H30B	0.9700	C6—C7	1.437 (4)
C31—H31A	0.9600	C9—C10	1.524 (4)
C31—H31B	0.9600	C9—H9A	0.9700
C31—H31C	0.9600	C9—H9B	0.9700
C27'—C28'	1.514 (2)	C10—C11	1.514 (4)
C27'—C19	1.527 (4)	C10—H10A	0.9700
C27'—H27C	0.9700	C10—H10B	0.9700
C27'—H27D	0.9700	C11—C12	1.520 (5)
C28'—C29'	1.527 (2)	C11—H11A	0.9700
C28'—H28C	0.9700	C11—H11B	0.9700
C28'—H28D	0.9700	C12—C13	1.523 (6)
C29'—C30'	1.519 (2)	C12—H12A	0.9700
C29'—H29C	0.9700	C12—H12B	0.9700
C29'—H29D	0.9700	C13—H13A	0.9600
C30'—C31'	1.564 (2)	C13—H13B	0.9600
C30'—H30C	0.9700	C13—H13C	0.9600
C30'—H30D	0.9700	C14—C18	1.344 (4)
C31'—H31D	0.9600	C14—C15	1.503 (3)
C31'—H31E	0.9600	C15—C16	1.536 (4)
C31'—H31F	0.9600	C16—C17	1.521 (4)
S1—C4	1.714 (3)	C17—C18	1.498 (3)
S1—C1	1.728 (3)	C18—C20	1.473 (4)
S2—C19	1.715 (3)	C19—C20	1.376 (4)
S2—C22	1.727 (3)	C20—C21	1.399 (4)
F1—C15	1.357 (3)	C21—C22	1.376 (4)
F2—C15	1.340 (3)	C21—H21	0.9300
F3—C16	1.341 (3)	C22—C23	1.421 (4)
F4—C16	1.344 (3)	C23—C24	1.345 (4)
F5—C17	1.356 (3)	C23—H23	0.9300
F6—C17	1.343 (3)	C24—C25	1.406 (5)
N1—C7	1.140 (4)	C24—C26	1.435 (5)
N2—C8	1.133 (4)		
			
C28—C27—C19	110.7 (2)	C4—C9—C10	112.9 (2)
C28—C27—H27A	109.5	C4—C9—H9A	109.0
C19—C27—H27A	109.5	C10—C9—H9A	109.0
C28—C27—H27B	109.5	C4—C9—H9B	109.0
C19—C27—H27B	109.5	C10—C9—H9B	109.0
H27A—C27—H27B	108.1	H9A—C9—H9B	107.8
C27—C28—C29	109.8	C11—C10—C9	113.5 (2)
C27—C28—H28A	109.7	C11—C10—H10A	108.9
C29—C28—H28A	109.7	C9—C10—H10A	108.9
C27—C28—H28B	109.7	C11—C10—H10B	108.9
C29—C28—H28B	109.7	C9—C10—H10B	108.9
H28A—C28—H28B	108.2	H10A—C10—H10B	107.7
C30—C29—C28	107.2	C10—C11—C12	113.6 (3)
C30—C29—H29A	110.3	C10—C11—H11A	108.8
C28—C29—H29A	110.3	C12—C11—H11A	108.8
C30—C29—H29B	110.3	C10—C11—H11B	108.8
C28—C29—H29B	110.3	C12—C11—H11B	108.8
H29A—C29—H29B	108.5	H11A—C11—H11B	107.7
C31—C30—C29	105.7	C11—C12—C13	112.8 (3)
C31—C30—H30A	110.6	C11—C12—H12A	109.0
C29—C30—H30A	110.6	C13—C12—H12A	109.0
C31—C30—H30B	110.6	C11—C12—H12B	109.0
C29—C30—H30B	110.6	C13—C12—H12B	109.0
H30A—C30—H30B	108.7	H12A—C12—H12B	107.8
C28'—C27'—C19	114.7 (3)	C12—C13—H13A	109.5
C28'—C27'—H27C	108.6	C12—C13—H13B	109.5
C19—C27'—H27C	108.6	H13A—C13—H13B	109.5
C28'—C27'—H27D	108.6	C12—C13—H13C	109.5
C19—C27'—H27D	108.6	H13A—C13—H13C	109.5
H27C—C27'—H27D	107.6	H13B—C13—H13C	109.5
C27'—C28'—C29'	113.3	C18—C14—C3	128.6 (2)
C27'—C28'—H28C	108.9	C18—C14—C15	110.7 (2)
C29'—C28'—H28C	108.9	C3—C14—C15	120.6 (2)
C27'—C28'—H28D	108.9	F2—C15—F1	106.4 (2)
C29'—C28'—H28D	108.9	F2—C15—C14	113.5 (2)
H28C—C28'—H28D	107.7	F1—C15—C14	111.3 (2)
C30'—C29'—C28'	112.6	F2—C15—C16	111.3 (2)
C30'—C29'—H29C	109.1	F1—C15—C16	109.8 (2)
C28'—C29'—H29C	109.1	C14—C15—C16	104.5 (2)
C30'—C29'—H29D	109.1	F3—C16—F4	107.2 (2)
C28'—C29'—H29D	109.1	F3—C16—C17	113.5 (2)
H29C—C29'—H29D	107.8	F4—C16—C17	109.5 (2)
C29'—C30'—C31'	108.2	F3—C16—C15	113.1 (2)
C29'—C30'—H30C	110.1	F4—C16—C15	109.4 (2)
C31'—C30'—H30C	110.1	C17—C16—C15	104.1 (2)
C29'—C30'—H30D	110.1	F6—C17—F5	105.5 (2)
C31'—C30'—H30D	110.1	F6—C17—C18	113.5 (2)
H30C—C30'—H30D	108.4	F5—C17—C18	110.2 (2)
C30'—C31'—H31D	109.5	F6—C17—C16	112.6 (2)
C30'—C31'—H31E	109.5	F5—C17—C16	110.0 (2)
H31D—C31'—H31E	109.5	C18—C17—C16	105.2 (2)
C30'—C31'—H31F	109.5	C14—C18—C20	128.9 (2)
H31D—C31'—H31F	109.5	C14—C18—C17	110.9 (2)
H31E—C31'—H31F	109.5	C20—C18—C17	120.0 (2)
C4—S1—C1	92.33 (12)	C20—C19—C27	130.1 (3)
C19—S2—C22	92.55 (14)	C20—C19—C27'	126.0 (3)
C2—C1—C5	124.0 (2)	C20—C19—S2	111.1 (2)
C2—C1—S1	110.45 (19)	C27—C19—S2	118.4 (2)
C5—C1—S1	125.4 (2)	C27'—C19—S2	122.2 (2)
C1—C2—C3	113.6 (2)	C19—C20—C21	112.4 (2)
C1—C2—H2	123.2	C19—C20—C18	123.2 (2)
C3—C2—H2	123.2	C21—C20—C18	124.3 (2)
C4—C3—C2	112.3 (2)	C22—C21—C20	114.1 (3)
C4—C3—C14	123.9 (2)	C22—C21—H21	122.9
C2—C3—C14	123.8 (2)	C20—C21—H21	122.9
C3—C4—C9	129.2 (2)	C21—C22—C23	124.6 (3)
C3—C4—S1	111.28 (19)	C21—C22—S2	109.7 (2)
C9—C4—S1	119.51 (18)	C23—C22—S2	125.6 (2)
C6—C5—C1	129.8 (3)	C24—C23—C22	129.8 (3)
C6—C5—H5	115.1	C24—C23—H23	115.1
C1—C5—H5	115.1	C22—C23—H23	115.1
C5—C6—C8	123.4 (3)	C23—C24—C25	123.0 (3)
C5—C6—C7	120.1 (3)	C23—C24—C26	121.2 (3)
C8—C6—C7	116.4 (3)	C25—C24—C26	115.8 (3)
N1—C7—C6	179.1 (4)	N3—C25—C24	179.7 (6)
N2—C8—C6	179.7 (4)	N4—C26—C24	178.4 (4)
			
C19—C27—C28—C29	−177.6 (3)	F4—C16—C17—F6	27.0 (3)
C27—C28—C29—C30	176.0	C15—C16—C17—F6	143.8 (2)
C28—C29—C30—C31	−172.5	F3—C16—C17—F5	24.5 (3)
C19—C27'—C28'—C29'	169.7 (3)	F4—C16—C17—F5	144.2 (2)
C27'—C28'—C29'—C30'	84.1	C15—C16—C17—F5	−98.9 (3)
C28'—C29'—C30'—C31'	97.4	F3—C16—C17—C18	143.1 (2)
C4—S1—C1—C2	0.8 (2)	F4—C16—C17—C18	−97.1 (2)
C4—S1—C1—C5	−175.5 (2)	C15—C16—C17—C18	19.7 (3)
C5—C1—C2—C3	174.4 (2)	C3—C14—C18—C20	−3.0 (5)
S1—C1—C2—C3	−1.9 (3)	C15—C14—C18—C20	174.8 (2)
C1—C2—C3—C4	2.4 (3)	C3—C14—C18—C17	−179.4 (2)
C1—C2—C3—C14	−176.1 (2)	C15—C14—C18—C17	−1.6 (3)
C2—C3—C4—C9	178.2 (3)	F6—C17—C18—C14	−135.4 (3)
C14—C3—C4—C9	−3.3 (4)	F5—C17—C18—C14	106.6 (3)
C2—C3—C4—S1	−1.7 (3)	C16—C17—C18—C14	−11.9 (3)
C14—C3—C4—S1	176.8 (2)	F6—C17—C18—C20	47.8 (3)
C1—S1—C4—C3	0.6 (2)	F5—C17—C18—C20	−70.2 (3)
C1—S1—C4—C9	−179.3 (2)	C16—C17—C18—C20	171.3 (2)
C2—C1—C5—C6	−174.7 (3)	C28—C27—C19—C20	124.9 (4)
S1—C1—C5—C6	1.1 (4)	C28—C27—C19—C27'	47.96 (12)
C1—C5—C6—C8	−2.9 (5)	C28—C27—C19—S2	−62.4 (3)
C1—C5—C6—C7	176.4 (3)	C28'—C27'—C19—C20	−174.6 (3)
C3—C4—C9—C10	121.1 (3)	C28'—C27'—C19—C27	−61.76 (13)
S1—C4—C9—C10	−59.0 (3)	C28'—C27'—C19—S2	15.3 (4)
C4—C9—C10—C11	−178.3 (2)	C22—S2—C19—C20	0.7 (2)
C9—C10—C11—C12	−178.7 (3)	C22—S2—C19—C27	−173.3 (3)
C10—C11—C12—C13	67.7 (4)	C22—S2—C19—C27'	172.2 (3)
C4—C3—C14—C18	−58.3 (4)	C27—C19—C20—C21	171.7 (3)
C2—C3—C14—C18	120.1 (3)	C27'—C19—C20—C21	−172.5 (3)
C4—C3—C14—C15	124.1 (3)	S2—C19—C20—C21	−1.4 (3)
C2—C3—C14—C15	−57.5 (4)	C27—C19—C20—C18	−5.5 (5)
C18—C14—C15—F2	135.8 (3)	C27'—C19—C20—C18	10.3 (5)
C3—C14—C15—F2	−46.2 (3)	S2—C19—C20—C18	−178.6 (2)
C18—C14—C15—F1	−104.1 (3)	C14—C18—C20—C19	−59.7 (4)
C3—C14—C15—F1	73.9 (3)	C17—C18—C20—C19	116.5 (3)
C18—C14—C15—C16	14.3 (3)	C14—C18—C20—C21	123.5 (3)
C3—C14—C15—C16	−167.7 (2)	C17—C18—C20—C21	−60.3 (4)
F2—C15—C16—F3	92.8 (3)	C19—C20—C21—C22	1.7 (3)
F1—C15—C16—F3	−24.7 (3)	C18—C20—C21—C22	178.8 (2)
C14—C15—C16—F3	−144.2 (2)	C20—C21—C22—C23	178.9 (3)
F2—C15—C16—F4	−26.5 (3)	C20—C21—C22—S2	−1.1 (3)
F1—C15—C16—F4	−144.1 (2)	C19—S2—C22—C21	0.2 (2)
C14—C15—C16—F4	96.4 (3)	C19—S2—C22—C23	−179.8 (3)
F2—C15—C16—C17	−143.4 (2)	C21—C22—C23—C24	−176.5 (3)
F1—C15—C16—C17	99.0 (3)	S2—C22—C23—C24	3.6 (5)
C14—C15—C16—C17	−20.5 (3)	C22—C23—C24—C25	1.2 (6)
F3—C16—C17—F6	−92.8 (3)	C22—C23—C24—C26	−178.3 (4)

## References

[bb1] Bruker (1997). *SAINT* Bruker AXS Inc., Madison, Wisconsin, USA.

[bb14] Bruker (2004). *APEX2* Bruker AXS Inc., Madison, Wisconsin, USA.

[bb2] Gilat, S. L., Kawai, S. H. & Lehn, J.-M. (1993). *J. Chem. Soc. Chem. Commun.*, pp. 1685–1716.

[bb3] Gilat, S. L., Kawai, S. H. & Lehn, J.-M. (1995). *Chem. Eur. J.* pp. 275–284.

[bb4] Irie, M. (2000). *Chem. Rev.* **100**, 1685–1716. 10.1021/cr980069d 11777416

[bb5] Kobatake, S., Kuma, S. & Irie, M. (2004). *Bull. Chem. Soc. Jpn*, **77**, 945–951.

[bb6] Pu, S.-Z., Yang, T.-S., Wang, R.-J. & Xu, J.-K. (2005). *Acta Cryst.* E**61**, o4077–o4079.

[bb7] Pu, S.-Z., Zhang, F.-S., Fan, S., Wang, R.-J., Zhou, X.-H. & Chan, S.-K. (2003). *Tetrahedron Lett.* **44**, 1011–1015.

[bb8] Sheldrick, G. M. (1996). *SADABS* University of Göttingen, Germany.

[bb9] Sheldrick, G. M. (2008). *Acta Cryst.* A**64**, 112–122. 10.1107/S0108767307043930 18156677

[bb10] Tian, H. & Yang, S.-J. (2004). *Chem. Soc. Rev.* **33**, 85–97.

[bb11] Woodward, R. B. & Hoffmann, R. (1970). *The Conservation of Orbital Symmetry*, pp. 98–100. Weinheim: Verlag Chemie GmbH.

[bb12] Yamaguchi, T. & Irie, M. (2006). *Bull. Chem. Soc. Jpn*, **79**, 951–1100.

[bb13] Zheng, C.-H., Pu, S.-Z., Le, Z.-G., Luo, M.-B. & Huang, D.-C. (2007). *Acta Cryst.* E**63**, o2578.

